# Applications of Multimodal Artificial Intelligence in Non-Hodgkin Lymphoma B Cells

**DOI:** 10.3390/biomedicines12081753

**Published:** 2024-08-05

**Authors:** Pouria Isavand, Sara Sadat Aghamiri, Rada Amin

**Affiliations:** 1 Department of Radiology, School of Medicine, Zanjan University of Medical Sciences, Zanjan 4513956184, Iran; 2 Department of Biochemistry, University of Nebraska, Lincoln, NE 68503, USA

**Keywords:** multimodal AI, non-Hodgkin lymphoma, AI, tumor microenvironment

## Abstract

Given advancements in large-scale data and AI, integrating multimodal artificial intelligence into cancer research can enhance our understanding of tumor behavior by simultaneously processing diverse biomedical data types. In this review, we explore the potential of multimodal AI in comprehending B-cell non-Hodgkin lymphomas (B-NHLs). B-cell non-Hodgkin lymphomas (B-NHLs) represent a particular challenge in oncology due to tumor heterogeneity and the intricate ecosystem in which tumors develop. These complexities complicate diagnosis, prognosis, and therapy response, emphasizing the need to use sophisticated approaches to enhance personalized treatment strategies for better patient outcomes. Therefore, multimodal AI can be leveraged to synthesize critical information from available biomedical data such as clinical record, imaging, pathology and omics data, to picture the whole tumor. In this review, we first define various types of modalities, multimodal AI frameworks, and several applications in precision medicine. Then, we provide several examples of its usage in B-NHLs, for analyzing the complexity of the ecosystem, identifying immune biomarkers, optimizing therapy strategy, and its clinical applications. Lastly, we address the limitations and future directions of multimodal AI, highlighting the need to overcome these challenges for better clinical practice and application in healthcare.

## 1. Introduction

In modern oncology, the clinical information per patient has significantly expanded, supplying cancer management with a comprehensive understanding of each patient’s unique tumor profile. The extensive use of high-dimensional data in clinical routines such as omics, imaging, and electronic clinical records leads to a deeper investigation of tumor heterogeneity that drives cancer progression and influences treatment response. Artificial intelligence (AI) has become essential in integrating large data scales to picture tumor behavior and treatment response [1,2,3]. A state-of-the-art approach called multimodal AI is an advanced form of AI that processes and integrates data from multiple sources or modalities including imaging, biological information, clinical records, and laboratory results, to create a comprehensive system of the disease [2,3,4]. In the realm of precision oncology, where treatments are tailored to the molecular makeup of patients, multimodal AI can act as an intelligent assistant to physicians, providing critical insights into tumor complexity and potential therapeutic targets [5,6]. AI, for instance, can uncover subtle genetic mutations and molecular alterations that drive tumor growth, which might be missed when using a single source [7]. Through multiple and complex data fusion, AI can discover intricate relationships within disparate data, predict disease progression, and stratify patients based on their risk profiles. Therefore, treatment regimens can be tailored to the unique characteristics of each patient, and efficiently support tracking of disease and response to therapy [8,9]. As disease and/or treatment evolve, patients are often monitored over time, leading to the accumulation of vast amounts of data that require effective strategies for integration and interpretability [10]. In this context, multimodal AI can help manage and support the integration of data over long time periods. This longitudinal approach supports the detection of subtle changes in a patient’s condition, facilitating early intervention and personalized adjustments to treatment plans [11,12]. Furthermore, integrating multi-dimensional data through AI can lead to the identification of novel tumor biomarkers and the development of innovative assays for cancer characterization, diagnosis, prognosis, and treatment planning [13]. Thus, multimodal AI ultimately holds the potential to transform the treatment of cancer, resulting in better patient outcomes and more effective healthcare delivery [14,15].

The use of multimodal AI in the case of B-cell non-Hodgkin lymphomas (B-NHLs) exemplifies the transformative potential of integrating diverse data sources in cancer research [16,17,18]. The non-Hodgkin lymphoma B cells (B-NHLs) represent a diverse spectrum of cancers originating from B lymphocytes, essential immune system cellular components. The most prevalent are diffuse large B-cell lymphoma (DLBCL) and follicular lymphoma (FL), which have been highly documented in modern research [19,20,21]. Both within subtypes and across patients the B-NHLs differ in severity, treatment response, and prognosis, complicating therapeutic strategies. Late diagnosis and relapse further contribute to the complexity of managing lymphoma cases [22,23,24]. Generally, B-NHLs are treated with a combination of therapies based on the disease subtype, stage, and patient health. The frontline regimen to treat B-NHLs is the combination of four chemotherapy drugs known as CHOP (cyclophosphamide, doxorubicin, vincristine, and prednisone) or combined with monoclonal antibody rituximab (R-CHOP), targeting B cells. Due to the complexity of the disease and patient response to conventional treatment, additional treatments involve antibody-based therapies, antibody–drug conjugate (ADC) with a cytotoxic agent, radiation therapy, and targeting signaling components of B cells such as ibrutinib, PI3K inhibitors, and venetoclax [25,26]. High-dose chemotherapy followed by stem cell transplantation is an alternative for instances that are aggressive or have relapsed [27,28]. The main obstacles in managing B-NHLs are identifying the subtype, choosing the best course of treatment, and dealing with the possibility of recurrence. Moreover, severe adverse effects from strong treatments, including immunotherapy, chemotherapy, and stem cell transplants, are frequently experienced by patients [29,30,31].

In recent years, lymphoma therapeutic research has included the tumor microenvironment (TME) to comprehend B-NHL complexity. The TME forms a protective niche in support of B-NHLs, fueling disease progression, immune evasion, and therapy resistance [32,33]. The TME is made up of complex cellular components such as stromal cells, immune cells (such as T cells and myeloid cells), as well as molecular factors, including extracellular matrix, and signaling, metabolic, and epigenetic networks [19,34,35,36]. Consequently, therapeutic strategies targeting TME have emerged, promising therapeutic methods to target tumor cells by harnessing the patient’s immune system. These approaches include immunomodulatory agents, such as checkpoint inhibitors, bispecific antibodies, and chimeric antigen receptor (CAR) T-cell therapy [37,38]. Additionally, small-molecule inhibitors targeting signaling pathways within the TME offer novel avenues for targeting molecular mechanisms that drive tumor growth and progression [39,40]. These strategies can potentially overcome resistance mechanisms in B-NHLs and improve current treatments and resistance therapy. However, long-term remission is still difficult to achieve despite advances in treatment strategies. This highlights the need for innovative approaches, such as multimodal AI, to address the complexity of lymphoma B cells and their ecosystem [41,42,43]. In this narrative review, we first define multimodal AI and its multiple modalities and support their usage by providing several applications in B-NHLs. We also discussed the limitations of multimodal AI in B-NHL investigations.

## 2. Multimodal AI

Multimodal AI is a field of AI that focuses on integrating and analyzing multiple data types from different sources and modalities. The objective of multimodal AI is to achieve more insights and understanding of cancer behavior than could be obtained from a single sort of data analysis alone [2]. Advanced AI systems such as machine learning (ML) and deep learning (DL) can effectively integrate and process these diverse data types to uncover complex patterns related to cancer. Machine learning, which is a subset of AI, involves using algorithms to allow computers to learn from and make predictions based on data. Deep learning is a subset of ML that primarily uses neural networks to analyze and interpret complex patterns in large datasets [2].

**Figure 1:**
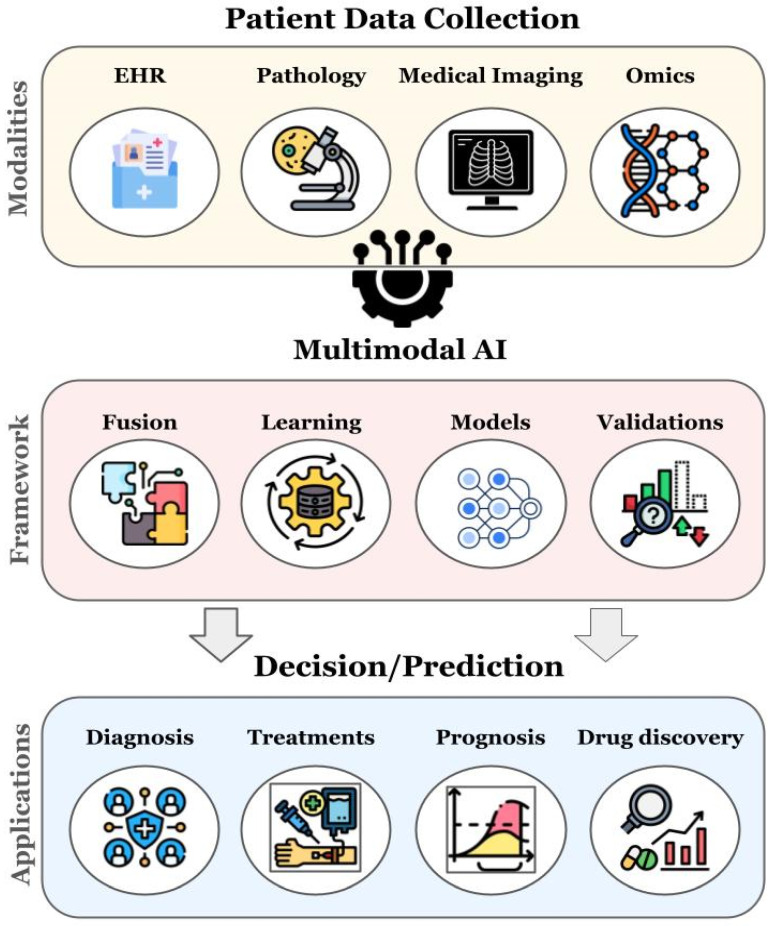
AI-driven multimodal biomedical data integration models, from patient data collection to the decision-making processes.

The multimodal AI approach is increasingly employed in healthcare, particularly in clinical decision-support systems. Historically, ML and DL applications in healthcare have focused on single-modal data. However, the biomedical field has been combining various data types through multimodal ML to enhance predictive accuracy and emulate the multimodal nature of clinical expert decision-making. For example, these modalities can include medical imaging, genomics, transcriptomics, proteomics, metabolomics, epigenomics, and electronic health records (EHRs) [17,44]. Figure 1 is a schematic representation of a multimodal framework for medical and biomedical data integration.

### 2.1. Different Types of Data Modalities

#### 2.1.1. Imaging Modality

While radiography may not be the most comprehensive imaging modality, it has the potential to identify soft-tissue masses in the chest, pharyngeal, and retropharyngeal regions, and even osseous involvement. Ultrasound (US), computed tomography (CT), and magnetic resonance imaging (MRI) have emerged as the dominant modalities for anatomic imaging in NHL [45,46,47]. MR diffusion-weighted imaging (DWI) detects the Brownian motion of water molecules within and between cells. In NHL tumors, with their high cellular density, this motion is restricted, leading to high signal intensity on DWI images and low signal intensity on the corresponding apparent diffusion coefficient (ADC) map [48]. In managing fluorodeoxyglucose (FDG)-avid NHL, PET/CT has emerged as a standard imaging modality for both initial disease staging and post-treatment response assessment [49,50]. In the evaluation of non-FDG-avid lymphomas, CT imaging retains its primacy. Furthermore, CT remains a valuable tool for targeted surveillance imaging in select clinical scenarios [51]. Whole-body MRI with short inversion time inversion recovery (STIR) sequences has emerged as a valuable tool for staging lymphoma, demonstrating high concordance with FDG PET/CT in disease detection [52].

Definitively diagnosing lymphoma depends on a meticulous microscopic examination of tissue sections by a trained pathologist. This analysis, conducted at various magnifications, relies on the characteristic morphological features revealed by hematoxylin and eosin (H&E) staining to guide the diagnostic formulation [53]. Advancements in whole-slide imaging (WSI) have fueled a recent shift toward digital pathology. Digital pathology involves creating high-resolution digital images from glass slides. This offers advantages, such as faster turnaround times, easier data management, remote access, and potentially more objective analysis [13].

#### 2.1.2. Medical Records

Electronic health records (EHRs) are digital archives of a patient’s medical history, integrating longitudinal data like demographics, diagnoses, clinical notes, and objective findings. This comprehensive view of a patient’s health journey fosters informed treatment decisions and improved care coordination [54]. EHRs integrate a broad spectrum of data types. This spectrum includes structured data, such as medication orders, with precise dates and dosages captured through a standardized electronic prescribing (e-prescribing) system. EHRs also incorporate unstructured data, exemplified by clinical notes that detail the clinical rationale underlying treatment decisions [55].

#### 2.1.3. Multi-Omics Data

Omics data include genomic, transcriptomic, proteomics, metabolomics, and epigenomics, which correspond to the analyses of genes, RNA, proteins, metabolites, and chromatin remodeling, respectively [56,57].

Genomic data help to identify driver mutations through analyzing DNA methylation, gene expression profiles, and mutations [58]. Transcriptome data shows how genes and RNA are active and regulated, revealing how cells dynamically use genetic information [59,60]. Epigenetics data explore chromatin accessibility, which is commonly disrupted in cancer and often causes abnormal expression of oncogenes and tumor suppressors [61,62]. Proteomics studies the full range of protein activities translated from the genome, closely linked with the epigenome and transcriptome [63]. Metabolomics offers a complementary perspective identifying major metabolic pathways that ecosystems rely on for growth and survival [64].

While a single modality can provide useful clinical information, adding multiple modalities enhances the accuracy of comprehending the fundamental biological functions of a tumor cell. This comprehensive approach goes beyond the limitations of individual data sources, facilitating a better understanding of cancer heterogeneity and patient-specific responses [65].

### 2.2. The Multimodal AI Framework

AI is pivotal in multimodal data fusion approaches for cancer research and treatment [65,66]. These algorithms extract and integrate quantitative information from diverse data modalities, to accurately segment tumors, classify them, and assess treatment responses [67]. AI-driven data fusion is versatile across various domains. For example, it can analyze integrated genomic and transcriptomic data to identify driver mutations, infer molecular subtypes, and predict survival outcomes [1,4,16,68]. Combining proteomic with genomic data and clinical information allows AI to identify therapeutic targets, assign risk level, and reveal additional driver mutations [4,68]. AI algorithms using pharmacological data predict drug responses, optimize treatments, and provide insights into drug interactions and efficacy for personalized therapeutic strategies [69]. In this section, we explained the multimodal AI framework for clinical and biomedical data integration to present how it enhances our understanding of tumor biology and supports more effective precision medicine.

### 2.3. Data Fusion Strategies

**Figure 2:**
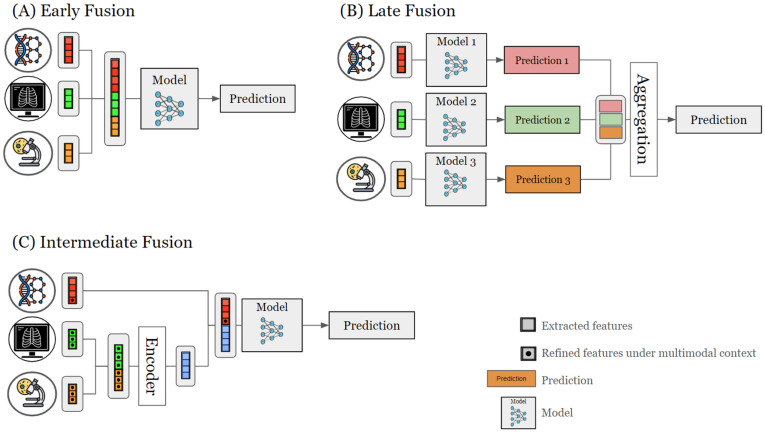
Data fusion strategies for multimodal AI framework. (**A**) Early fusion integrates multiple modalities at the beginning of the learning process, as illustrated in the figure with examples such as omics, medical images, and pathology data. Each modality undergoes independent feature extraction to derive distinct features. These extracted features are then combined through concatenation, forming a unified representation. This fused representation serves as the input to a single model, which is trained to predict the target variable. (**B**) Late fusion processes each modality independently until the final prediction stage. The figure presents three individual models, each trained on a different modality. These models generate individual predictions, which are then aggregated to form a final prediction. (**C**) Intermediate fusion strategy involves combining features from different modalities at an intermediate stage of the learning process. The figure shows a shared encoder that combines the features from different modalities into a unified representation. The multimodal model propagates gradients back to the feature extraction layers of each modality to refine feature representations within the context of the other modalities, enhancing the overall performance and learning process. This representation is then processed by a single model to generate a final prediction.

Multimodal AI frameworks employ various data fusion methods to integrate various data modalities, each method with unique advantages and challenges. Here, we address three categories of fusion strategies (Figure 2) used in cancer multimodal AI studies, including early, late, and intermediate data fusion [2,44,69].

#### 2.3.1. Early Fusion

Early fusion, or data-level fusion, combines multiple data sources into a unified information space before analysis [70]. This method trains a single model using quantitative features extracted jointly across modalities, capturing interactions at a low level to reveal subtle patterns. It promotes a holistic understanding of data, especially when inter-modal relationships are critical [2]. By integrating data at the input level, early fusion potentially improves processing efficiency and reduces computational complexity later on [44]. However, integrating diverse data types can be challenging due to differences in data scale, dimensions, or sample rates [2]. This challenge may require complex preprocessing to align data properly [2,13], as incomplete or noisy datasets can affect the analysis pipeline, reducing robustness and introducing biases in model performance.

#### 2.3.2. Late Fusion

Late fusion in multimodal data integration involves processing each modality independently to train separate models, then combining their predictions or representations at a later stage [70]. This method maximizes the extraction of relevant features from each data type, allowing for customized models for each modality. This approach can be particularly beneficial for handling diverse data sources that require different processing techniques [2]. Late fusion enhances robustness to missing or corrupted data in individual channels, which is crucial in real-world applications where data integrity cannot always be guaranteed across all modalities [2]. However, late fusion may struggle to capture subtle interactions between modalities, potentially missing important patterns that early fusion integrates from diverse data types [2,13]. This limitation can result in a less comprehensive understanding of complex phenomena that require integrated analysis of heterogeneous data [13,71,72]. Additionally, managing separate models for each modality in late fusion can increase computational demands, especially with large-scale datasets or limited resources.

#### 2.3.3. Intermediate Fusion

Intermediate fusion, also known as hybrid fusion, combines data modalities during the learning process at an intermediate stage. This method involves processing each modality’s attributes individually, and then, integrating them at a higher level of abstraction [2]. Intermediate fusion offers a compromise between early and late fusion by (i) facilitating the integration of low- and high-level features, (ii) capturing a wide range of inter-modal interactions at various levels of abstraction, and (iii) offering a greater flexibility in the data fusion design. This approach leads to more comprehensive and nuanced analyses [2] and remains closer to the unique properties of data, possibly balancing between integrated and modality-specific processing [2,13]. However, the multi-stage nature of intermediate fusion may necessitate more advanced engineering techniques and larger computational resources due to difficulties in model design, implementation, and optimization. The additional parameters and integration stages in intermediate fusion models may increase the risk of overfitting, especially with limited training data [2]. Therefore, rigorous validation techniques and model regularization are essential to ensure robust performance and generalizability.

Overall, the choice of an optimal fusion method for multimodal AI depends on several factors: (i) the objectives of the analysis, (ii) the nature and compatibility of the data sources, and (iii) the complexity of the fusion algorithm. The most suitable fusion approach can be limited by data redundancy between modalities, missing or noisy data, and the scalability of the method for large datasets. Despite these challenges, implementing appropriate fusion algorithms allows multimodal AI systems to effectively utilize complementary information from multiple modalities, leading to enhanced performance, more comprehensive analysis, and improved decision making.

### 2.4. Learning Methods

Predictive models are constructed by learning from a dataset, to make decisions based on identified patterns and relationships within the data. The training dataset typically includes an input, often represented as a set of features, and a corresponding output, known as a label. These models can be trained using three primary learning paradigms: supervised learning, unsupervised learning, and semi-supervised learning [73].

#### 2.4.1. Supervised Methods

Supervised learning is a fundamental paradigm in AI as it involves training models with labeled data, where each example includes inputs (often from multiple sources) and corresponding outputs (e.g., diagnostic labels). This method teaches models to learn associations between input modalities and their associated outcomes, making predictions on new, unlabeled data. When datasets are labeled, supervised learning is particularly valuable for multimodal AI applications, facilitating the discovery of relationships between diverse data modalities and their corresponding results. By mapping inputs to desired outputs, supervised learning improves the accuracy of tasks like classification, regression, and prediction, ultimately improving model performance and the interpretation of complex medical information [74].

#### 2.4.2. Unsupervised Methods

Unsupervised learning operates on unlabeled data to identify latent patterns and structures within the dataset. This learning method is particularly valuable in multimodal AI for identifying hidden correlations across distinct data modalities, especially when labeled information is missing. Common strategies employed in unsupervised learning for multimodal environments include (i) generative modeling, which seeks to learn the underlying data distribution and generate new data points; (ii) clustering, which groups similar data points together based on their inherent characteristics; and (iii) dimensionality reduction, which simplifies complex data by reducing the number of features while preserving essential information [75].

#### 2.4.3. Semi-Supervised Methods

Lastly, semi-supervised learning bridges the gap between fully supervised and unsupervised techniques by leveraging partially labeled or noisy data for training. This approach is particularly useful when acquiring complete labeled data is difficult or expensive. For instance, semi-supervised learning techniques in medical imaging can be employed when pixel-level annotations are unavailable and only image-level labels are accessible. By utilizing the available weak annotations, these methods aim to extract meaningful representations, identify hidden patterns across modalities, and ultimately train models to perform accurate predictions despite the limited labeled information [76].

In summary, selecting an appropriate learning paradigm, supervised, unsupervised, or semi-supervised, for multimodal AI applications depends on factors such as the availability and nature of the data and the desired outcomes. Supervised learning, utilizing labeled data, excels at identifying associations between input modalities and their corresponding outcomes, while unsupervised learning, operating on unlabeled data, is adept at uncovering hidden patterns and structures. Semi-supervised learning bridges the gap, optimizing partially labeled or noisy data when complete labeling is not feasible. Each paradigm presents distinct advantages and use cases, contributing to developing effective and robust multimodal AI frameworks.

### 2.5. Application of Multimodal AI in Precision Medicine

#### 2.5.1. Liquid Biopsy

Liquid biopsies, unlike traditional tissue biopsies, emerge as early detection tools through minimally invasive procedures to analyze circulating biomarkers such as circulating tumor DNA, circulating tumor cells, extracellular vesicles, and other circulating molecules. The integration of AI in liquid biopsy screening can facilitate high-throughput mapping of genetic and molecular information [77,78]. In their study, Zhang et al. employed a multimodal AI approach for the early diagnosis of lung cancer. Their methodology involved fusing several modalities with an ML method and testing their performance both individually and in combination. The modalities included extracellular vesicle long RNA (evlRNA) from liquid biopsy, various CT features, and the expertise of junior or senior experts in tissue analysis. The overall performance was classified as follows: the data fusion of evlRNA, imaging features, and senior expert input outperformed all other combinations with an accuracy of 93.4%. This was followed by the combination of evlRNA, imaging, and junior expert input, achieving an accuracy of 92.4%. The third best-performing combination was evlRNA plus imaging, with an accuracy of 91.9%. The weakest accuracies were observed for evlRNA alone (79.2%) and imaging alone (77.6%). These findings suggest that combining liquid biopsy with imaging and expert assessments enhances predictive capability significantly [79].

#### 2.5.2. Immunotherapy

The tumor mutational burden (TMB) and the expression of the immune checkpoint PD-1 are primary biomarkers used to identify patients likely to respond to immunotherapy; however, their predictive power remains limited [80,81]. To address this limitation, the National Institute of Health (NIH) developed a logistic regression-based immunotherapy-response score (LORIS) model, creating a clinical score to stratify patients based on their expected response to immunotherapy. The model used six frequently measurable patient features to provide clinical scores based on neutrophil-to-lymphocyte ratio, age, cancer type, therapeutic history, albumin levels, and TMB. LORIS demonstrated a greater performance compared to TMB alone by effectively identifying patients who were previously considered poor candidates for checkpoint inhibitors. Overall, the multimodal aspect of LORIS based on clinical, pathologic, and genomic features enhances the ability to personalize immunotherapy treatments [82].

#### 2.5.3. Surgery

By leveraging multimodal data, AI can enhance standard methods, providing deeper insights from data integration. For example, surgery is one of the gold standard techniques in cancer treatment, often being the primary approach for removing and reducing cancer spread. Assessing the tumor size and mass is often estimated through traditional imaging, which is critical in the management of surgery. However, imaging showed limitations in detecting small metastasis and often underestimates the metastatic spread leading to poor detection of occult metastasis [83,84,85]. Therefore, evaluating these hidden metastatic niches can greatly improve surgery and patient outcomes. To tackle these challenges, an open trial (NCT06478368) is under investigation to advance the early detection of hidden metastases of gastric cancer based on a multimodal AI approach. The study will integrate AI with dynamic video recordings of the abdominal cavity during surgery, supplemented by imaging, histopathology, and clinical data. Ultimately, the study aims to develop a non-invasive, real-time diagnostic tool for early and accurate detection of peritoneal metastasis.

#### 2.5.4. Clinical Trials

Clinical trials are also exploring the concept of multimodal AI and expanding its application in cancer clinical research [86]. For example, the clinical trial NCT05426135 focuses on developing an AI system for evaluating tumor risk, diagnosis, and treatment based on multimodal data fusion using DL technology. The two main objectives of this observational investigation are first to establish a medical platform with clinical, imaging, pathological, and multi-omics data for lung, stomach, and colorectal cancers. Then, the second goal is to create accurate diagnosis and treatment prediction models using AI technology and DL methods.

Another investigation, NCT06241092, is focussing on exploring the molecular heterogeneity of papillary thyroid carcinoma and associated TME using an AI-based multimodal by using genomic, transcriptomic, and pathological images to predict lymph node metastasis (LMN) and survival outcome. In their study, the authors produced a heatmap highlighting the high-risk tumor regions based on DL methods, indicating significant accuracy when predicting disease free survival for 1, 3, and 5 years. In addition, they also showed that LMN is associated with macrophages, cancer-associated fibroblasts, and T cells, illustrating how multimodal AI can provide several biomarkers to enhance prognosis [87].

The future of multimodal AI in cancer research seems promising, with ongoing research focused on improving standard methods such as surgery, prognosis, and treatments. Collaborative efforts between researchers, clinicians, and technology developers will be crucial in overcoming current challenges and harnessing the full potential of AI to transform cancer treatment.

## 3. Case Studies of Multimodal AI in B-NHLs

The significance of multimodal AI depends on its ability to efficiently build customized systems or one-size-fits-all models based on patient profiles. This feature has proved valuable for B-NHLs, as the combination of many modalities improves the efficacy of treatment and the accuracy of diagnosis. Here, we have grouped several research cases where multimodal AI is used in lymphoma B cells.

### 3.1. Investigating Tumor B-NHLs and TME Ecosystem

The multimodal benefit lies in the improved precision and effectiveness of cancer identification and categorization. By combining data from different diagnostic modalities, multimodal AI can enhance the sensitivity and specificity of lymphoma stratification. For example, Loeffler-Wirth et al. present a comprehensive transcriptome map covering six lymphoma subtypes, including DLBCL and FL, providing insights into their gene expression patterns. Their study included clinical, pathological, genetic, and transcriptome data from a sizable dataset of 873 biopsy specimens. The self-organizing map (SOM) machine learning technique was employed to create a low-dimensional representation of high-dimensional data and visualize its distribution as a map. The SOM was trained to generate distinct portraits for every sample using the expression data. In response to overlaps between portraits of distinct subtypes, the authors identified pattern types, stratifying lymphomas based on combinations of over-expressed genes in each sample. The prominent patterns detected were associated with proliferation, inflammation, and stroma signatures. In addition, combinatorial pattern types revealed that lymphoma subtypes were arranged as a spectrum of molecular expression levels rather than having distinct phenotypes. The various co-expressed modules associated with different functional categories, genetic abnormalities, and lymphoma pathophysiology demonstrated that poor survival was correlated with proliferation, inflammation, and plasma cell features. In contrast, inflammatory and stroma signatures linked to healthy B cells and normal tonsils were associated with higher overall survival rates [88].

Xu-Monette et al. investigated the potential of integrating targeted next-generation sequencing and AI to develop a cell-of-origin (COO) classifier for DLBCL. The study aimed to improve upon existing COO classification methods and explore its predictive value for prognosis, risk assessment, and treatment response. Using a cohort of 418 DLBCLs, the authors developed a more advanced classifier for DLBCL categorization using transcriptomic and genomic data.

Univariate significance tests and the Benjamini–Hochberg false discovery rate were utilized to select relevant features from the data modalities. Then, autoencoders, an unsupervised learning method from neural networks, were applied for dimensionality reduction. These autoencoders performed nonlinear transformations of autoencoded features, transforming the selected features into a two-dimensional latent space. Logistic regression and Cox proportional hazards models were then utilized to build separate models for developing the COO model and predicting clinical risk, respectively. The classifier demonstrates strong agreement with accepted COO classification methods such as the Nanostring Lymph2Cx assay [89]. While the primary focus of the study was on developing a robust COO classifier, the model was also capable of predicting clinical outcomes. Their results indicated that the COO from activated B cell-like (ABC) subtypes had much shorter survival rates than the COO from the germinal center B cell-like (GC) type. The COO classifier indicated that 30% of the patient set was classified as high-risk patients with poor survival. These findings show that tumor stratification may be improved by integrating AI and next-generation sequencing for clinical practice and precision therapy [90].

Multimodal AI also provides a comprehensive understanding of the complexity of lymphoma–TME interaction, which is crucial given the vital role of TME in affecting treatment response [32,33]. Radtke et al. recently developed a cellular and molecular multiscale atlas to investigate the FL environment using a range of modalities, such as genomic, transcriptomic, clinical, and pathological imaging. The Kassandra algorithm deconvolves bulk RNA sequencing (RNA-seq) data to predict cell percentages. An adversarially regularized variational graph autoencoder was used to analyze the spatial relationships between cells in images. The goal was to identify cellular communities that exhibit similar spatial arrangements and marker expression patterns. A convolutional neural network (CNN) was used to segment individual cells in images. The CNN for object-based segmentation and cell typing in images, enabled accurate identification of individual cells across different imaging modalities for multimodal data fusion.

The authors sought to understand the mechanisms behind the disease’s development and treatment effects by investigating both changes within cells and external influences on FL patients. According to the molecular analysis, there is a notable increase in pathways associated with immune signaling activation, cytokine signaling, extracellular matrix remodeling, and B-cell receptor signaling in early relapsers. These signaling pathways are driven by the proximity between FL B cells and the niche composed of myeloid and stromal cells. The study also revealed unique follicular growth patterns that started twenty months before the initial relapse, suggesting early alterations linked to the advancement of the disease. This study highlights the intricate networks between tumor cells and the TME as FL progresses and proposes potential markers for high-risk FL patients who experience an early recurrence [91].

### 3.2. Immune Biomarker Discovery

Biomarker discovery in B-NHLs has been vital for advancing B-NHL diagnosis, prognosis, and treatment, as biomarkers related to genetic, epigenetic, and signaling alteration have improved understanding of the mutational landscape and TME crosstalk [19,92]. Similarly to drug discovery, multimodal AI might support the discovery of novel biomarkers and therapeutic immunomodulators associated with TME regulation.

Steen et al. created the EcoTyper ML platform to analyze the complex relationships between tumors and TME in this context. EcoTyper employs the ML technique to integrate transcriptome deconvolution and single-cell RNA-seq to characterize clinically relevant DLBCL cell states and ecosystems. After estimating cell type proportions in bulk tissue transcriptomes, a non-negative matrix factorization (NMF) is used to identify distinct cell states within each cell type, based on purified gene expression profiles. EcoTyper uses a community detection algorithm, based on Jaccard indices and hierarchical clustering, to define multicellular ecosystems or ecotypes, integrating information from both bulk and single-cell datasets.

This innovative approach aims to clinically characterize relevant cell states and cellular ecosystems of DLBCLs. The cellular information was gathered from bulk RNA sequencing of both cancerous and healthy tissues and cell states from single-cell transcriptome data. The platform can recognize five cell states connected to somatic subtypes and general survival through this approach. The model identified 39 TME cell states from main immunological (e.g., CD4, CD8, plasma cells, natural killer, mast cells, monocytes, dendritic cells, neutrophils) and stromal populations (e.g., fibroblast and endothelial cells). This comprehensive DLBCL atlas generated by EcoTyper enables the capture of DLBCL ecosystems and the clinical diversity within existing subtypes. This method adds additional understanding of tumor classification beyond traditional approaches based on COO and genotypic features, offering valuable insights for therapeutic opportunities [93].

Carreras et al. [94] conducted a comprehensive pan-cancer analysis to identify biomarkers by integrating gene expression and proteomic immunohistochemical data, specifically on infiltrated immune cells. The study employed a suite of ML techniques and two neural networks on a series of 233 DLBCL patients treated with R-CHOP therapy to identify prognostic factors and classify lymphoma subtypes with high accuracy. Based on a selection of 25 genes extracted from an extensive library of 54,614 gene probes, a neural network could precisely predict the prognosis for 100 cases of DLBCL. Furthermore, immune profiling enriched markers associated with tumor-associated macrophages, T lymphocytes, and regulatory T lymphocytes, offering potential therapeutic avenues through targeted immune cell inhibitors.

Although the authors addressed that each ML and artificial neural network analysis using gene expression data have some strengths and weaknesses, they ranked the overall accuracy of each algorithm. The top three methods were reported to be XGBoost tree, random forest, and random trees, with accuracies of 100%, 98.3%, and 97.1%, respectively. The ranking of accuracy based on each AI algorithm underscores the importance of selecting appropriate AI methods tailored to the dataset size, cohorts, and modalities employed. Overall, the data suggest the significance of immuno-oncology biomarkers in elucidating the immune response in lymphoma [94].

In their recent study, Krull et al. explored the diverse cellular characteristics of FL by pairing data modalities from 87 newly diagnosed or untreated FL biopsies and employing advanced AI techniques for B-cell gene clustering using NMF and the cophenetic correlation coefficient to evaluate the stability and reliability of clustering results. The authors connected each transcriptional state to particular genetic abnormalities and interactions within the microenvironment by combining transcriptomic, genome sequencing, and TME analysis. While three distinct transcriptional states (inflammatory, proliferative, and chromatin remodeling) were identified by transcriptomic analysis in FL B cells, the combination of genomic and TME profiling revealed enrichment with immune evasion and high T cell infiltration, and linked COO to a prior germinal center B phenotype. These findings provided profound insights into FL B-cell transcriptional states and the intricate crosstalk with the immune microenvironment [95].

### 3.3. Therapy Optimization

Considering the timeline and the cost to launch an anti-cancer drug into the market, leveraging multimodal AI might accelerate the process of drug discovery by combining information on drug properties, protein interactions, genetic factors, and clinical outcomes [96,97,98].

Yeh et al. constructed a comprehensive genetic and epigenetic network through extensive data mining and derived relevant signaling networks unique to each DLBCL subtype (ABC and GC).

Then, a deep neural network model was trained using a dataset of known drug–target interactions. The model was designed to learn complex relationships between drug and target features, encoded as numerical vectors, to predict the probability of interaction between a drug and a specific protein target. The AI drug–target interaction model projected drug candidates more likely to interact with the discovered biomarkers by considering drug–target interaction, drug regulation, and drug toxicity. The authors contributed to possible therapeutic approaches for ABC and GC subtypes by identifying five pathogenic biomarkers and proposed four FDA drug candidates for these aggressive lymphoma classes. The framework proposed by Yeh et al. can serve as a roadmap for future drug selection, specifically tailored to the molecular pathways unique to tumor subtypes [99].

Another advantage of multimodal AI is the potential to advance personalized lymphoma therapies by utilizing pre-existing clinical data. With the expansion of public databases for different modalities, the construction and training of models using pre-existing data offer non-invasive tools to address the heterogeneity of patient responses to therapy. For instance, the prognostic model LymForest-25 integrated transcriptome and clinical information to examine the effectiveness of including bortezomib in first-line chemotherapy regimens for DLBCL patients. The model is based on a random forest algorithm, which combines multiple decision trees to enhance prediction accuracy. The model was trained on a cohort of patients treated with R-CHOP, and then, used to predict survival outcomes in a separate cohort of patients treated with both bortezomib and R-CHOP. Incorporating bortezomib into the standard R-CHOP therapy regimen may reduce the risk of death or disease progression by 30% in individuals with high-molecular-risk DLBCL. Furthermore, the model highlights the utility of multimodal AI in evaluating treatment response and predicting outcomes in DLBCL patients [100].

A non-invasive AI method utilizing three clinical imaging modalities from FL and DLBCL patients treated with CD19 CAR T-cells before and after treatment was designed by Tong et al. Radiologic image analysis, utilizing CT, low-dose CT, and 18F-FDG-PET, predicted patient-level lesion response to chimeric T cell treatment. Patients were categorized as treatment responders or non-responders based on lesion characteristics such as size or metabolic activity utilizing AI for lesion-level prediction. The study used DL-based image analysis methodology, trained on radiologic images, and then, the model applied a decision-making process known as rule-based reasoning to predict the lesion level in lymphoma patients. The integration results were then compared to the performance of the traditional International Prognostic Index (IPI) in a testing cohort of 26 patients with DLBCL, comprising 10 responders and 16 non-responders. Despite the small cohort, the analysis demonstrated promising accuracy in predicting treatment response at both lesion and patient levels, surpassing IPI [101].

A study led by Hong Lee et al. used clinical data and digital pathology pictures to create a predictive model from a cohort of 216 DLBCL patients treated with different chemotherapy and immunochemotherapy regimens. In this study, the authors described a sophisticated approach where they initially extracted features from tissue slides using self-supervised contrastive learning with the self-distillation with no labels (DINO) method, a technique that learns from unlabeled data. They then employed a dual-stream multiple-instance learning network with attention-based pooling to process image patches effectively and assign scores that highlighted important regions within the input data. This approach aimed to enhance the model’s ability to understand and utilize complex patterns in the data without requiring labeled annotations, a crucial aspect given the limited availability of labeled histopathology images for DLBCL. The multimodal predictive model was developed to evaluate drug response in DLBCL patients. Notably, the model was strongly associated with established clinical factors such as IPI and successfully predicted both immunotherapy response and relapse-free survival. These results indicate the possibility of using these approaches as supplementary prognostic instruments [102].

These clinical studies are examples of various methodologies to create non-invasive tools to investigate possible treatment options. Therefore, multimodal AI can support clinicians in therapeutic strategies and drug discovery by helping with multiple steps, from target identification and drug selection to the clinical outcome of the treatments.

The multimodal AI approaches discussed in this section show that AI algorithms can reveal biological insights, hidden patterns, and connections that single modality analysis techniques could miss. This can drive advances in precision medicine and drug development by identifying new biomarkers for prognosis, early detection, and possible biological targets for novel therapeutic strategies.

### 3.4. Clinical Investigations

Non-invasive approaches are a prevalent category in AI applications evaluated in clinical trials.

For instance, NCT04154228 is a prospective study aimed at developing an AI-based tool to measure early treatment response and differentiate between benign and tumorous masses in lymphoma patients. This non-invasive AI approach is built on the collection of clinical records, FDG PET/CT and PET/MR scans at various time points: before initial therapy, after 2/3 treatment cycles, and at the end of treatment to stratify lymphoma patients and evaluate high-risk patients.

The SYNERGY-AI Registry (clinical trial NCT03452774) is an international, prospective, observational cohort study designed to evaluate the clinical utility of AI in precision oncology. The study is testing the feasibility of AI-based tools to enhance decision making in clinical trial enrollment (CTE) for patients with advanced cancer. The study is collecting EHRs and genetic mutation data, targeting a diverse group of adult and pediatric patients with advanced solid and hematological malignancies, including B-NHLs. Over a period of 36 months, including 24 months of patient enrollment and 12 months of data collection, the study will assess the time to initiation of CTE, progression-free survival, and overall survival. The purpose of the study is to maximize trial enrollment by using ML algorithms to match patient clinical data with ongoing clinical studies.

Overall, these examples demonstrate the complementary nature of integrating multimodal AI in B-NHLs to enhance decision making, leveraging diverse data sources such as genetic profiles, imaging modalities, and clinical parameters to provide more comprehensive and personalized treatment strategies.

## 4. Limitations and Challenges

Although multimodal AI has great potential for B-NHL diagnosis and treatment, proper representation of the disease biology depends heavily on data quality and availability, advanced AI techniques for data fusion and interpretability of results, and robust validation methodologies.

### 4.1. Data Quality and Availability

#### 4.1.1. Omics

Variations in sample collection, storage, and processing can affect the reliability of the data. The preservation techniques used for tissues and the interval between sample collection and analysis can impact the quality of biological extraction, especially for omics data [103,104,105]. Moreover, data variability can also be enhanced by selecting analytical platforms, like sequencing technologies, which can lead to variations in sensitivity and specificity [106,107]. These differences are further exacerbated by inconsistent data preprocessing and normalization methods used in different studies, which makes it difficult to compare outcomes and reach robust conclusions [108,109]. Thus, it is essential to develop standardized procedures for multi-omics analysis and sample handling to reduce sources of variability and guarantee high-quality, repeatable findings across various investigations.

Access to large and diverse datasets is fundamental to the development and functionality of multimodal AI models. Without sufficient and varied data, these models will fail to capture the whole tumor biology and may exhibit biases or limitations in their predictions. In addition to data availability, the compatibility of merging data with similar biological characteristics from different sources presents additional challenges, requiring robust data integration and preprocessing techniques [2]. Additionally, data collection, curation, and annotation are often costly, resource-intensive, and time consuming, emphasizing the importance of readily available and curated datasets [110,111].

#### 4.1.2. Imaging

Preparing and staining tissue sections for pathology imaging is vital for obtaining high-quality images. Key steps like tissue collection, fixation, paraffin embedding, sectioning, antigen retrieval, and staining are crucial for preserving molecular and cellular integrity, essential for accurate immunohistochemical staining [112]. For example, tissue blocks are subjected to heat-induced epitope retrieval (HIER) or enzymatic digestion to expose antigens for staining, but excessive heat can damage antigens and cause staining artifacts. Moreover, the staining process relies on precise antigen–antibody recognition, which must be carefully assessed individually for each antigen and antibody post-HIER to ensure accurate binding specificity and sensitivity. Additionally, the lack of standardization methods between labs means ML and DL algorithms trained on data from one center may not perform well on data from another, limiting their generalizability [113]. Therefore, establishing standardized imaging methods is crucial for consistency and reproducibility across centers, especially when integrating these images into multimodal AI systems for comprehensive cancer analysis.

Radiology imaging is essential for medical diagnostics but presents limitations that the multimodal AI framework has to consider [114]. Certain steps are required to be taken before medical images can be used for the development of an AI algorithm. One of the radiology AI research challenges is the standardization of data regarding data heterogeneity [115]. Data from multiple institutions across different geographic regions would encompass a diverse range of imaging equipment, ethnicities, and pathologies; however, single-institution data are often used due to limited access to multi-institutional datasets [115]. Also, the quality of an image can be affected by factors like patient movement, image acquisition settings, and post-processing techniques, which can reduce interpretability of data [116,117]. However, harmonizing data collected across diverse healthcare institutions is challenging. Most of the AI models are trained on local datasets that exhibit relative homogeneity in their patient populations. Such datasets can be susceptible to bias introduced by institutional factors, including manufacturer preferences for imaging equipment, established protocols for data acquisition, and variations in medical staff training [118,119].

Overall, multimodal AI methods must be robust to handle diverse, large-scale data and ensure compatibility when combining multiple imaging. This robust integration is crucial for providing comprehensive insights that support clinical decision making.

### 4.2. AI Methods and Strategies for Data Fusion

Selecting AI methods affects both interpretability and model development. Because of their computational complexity, complex datasets require large data processing capacity and advanced algorithms for preprocessing and analysis. DL algorithms, widely used as AI models, function as “black boxes”, making it difficult to comprehend how they arrive at particular conclusions [120]. Biases in the data used to train AI algorithms may result in less accurate predictions for particular conditions or populations. Several sample datasets must be used to train models to improve accuracy and generalizability for the clinical interpretation [120,121].

Determining the best approach for combining several modalities to create the model is another factor to consider when utilizing AI, as it can directly affect the accuracy and performance of the model [13]. There is no “one-size-fits-all” method for the various examples of multimodal AI strategies employed in B-NHLs; instead, the best fusion strategy depends on the question addressed, the dataset, and clinical expertise. For example, an image scan requires a radiologist for tumor segmentation and clinical validation. Moreover, determining when to fuse modalities involves addressing several challenges. There may be discrepancies in the temporal or spatial alignment of data from different modalities, making it challenging to synchronize and integrate them effectively [2]. Additionally, the heterogeneity and complexity of multimodal data can pose difficulties in identifying meaningful patterns and relationships across modalities [122,123]. Selecting the most appropriate fusion strategy requires careful consideration of the trade-offs between computational complexity, interpretability, and the ability to capture synergistic information from different modalities [71]. Overall, the absence of best practices and standardized procedures for multimodal fusion adds additional difficulties in sorting through the many fusion methods and deciding when and how best to merge modalities.

### 4.3. Clinical Implementation

The transition from multimodal AI to clinical implementation may represent a challenge in translating research findings into real-world clinical settings. Currently, AI is incorporated into medical devices and routine healthcare procedures. Healthcare practitioners frequently use these devices to help with diagnosis, treatment planning, patient monitoring, and other medical duties. As of March 2024, the US Food and Drug Administration (FDA) reports that 882 medical devices with AI/ML capabilities across various medical specialties have been approved for usage in the USA. Of these, radiology is particularly well represented, making up a sizable portion (80%) [124,125].

Despite the promising implementation of AI in healthcare, several limitations must be overcome before a clinical multimodal AI model can be integrated into clinical practice. First, no regulatory frameworks or standards exist for assessing and approving multimodal AI algorithms for clinical usage [126,127]. Because of this uncertainty, healthcare professionals may be reluctant to employ algorithms in clinical settings until they have received official validation and regulatory approval [128,129]. Second, logistical and financial challenges arise when integrating multimodal AI technologies into clinical workflows. Its implementation in clinical settings can be time consuming and will require substantial financial investment for testing, validation, regulation, and training healthcare personnel. The production of high-dimensional data for multimodal data integration will require initial investment in equipment, software, and infrastructure to handle large volumes of diverse data types efficiently. Then, ongoing costs for upgrades, and data management systems further contribute to the financial demands of implementing and sustaining multimodal AI frameworks in clinical settings [110,111]. In addition, testing AI algorithms can be costly due to the need for extensive validation processes, which require storage of large-scale data, computational resources, and specialized expertise to ensure robust performance and reliability. The list of financial burdens also includes the recruitment and training of healthcare professionals in AI applications and ensuring compliance with regulatory standards. Due to the complexity and heterogeneity of healthcare systems, integrating new multimodal technologies will frequently necessitate considerable staff training, processes, and infrastructure. Despite these initial costs, the long-term benefits of improved patient care and operational efficiency underscore the value of investing in multimodal AI for healthcare [130]. Lastly, there are ethical and legal considerations surrounding the use of AI in healthcare, particularly concerning patient privacy, data security, and liability [131]. The growing digital data repositories help provide easy access to patient data for telemedicine and consultation, therefore (i) enhancing the accuracy of diagnoses, (ii) improving patient monitoring, and (iii) optimizing the overall healthcare delivery process. They also raises the risks of clinical record leaks and illegal access to confidential medical data [131]. Ensuring transparency in the use of AI in healthcare is morally a requirement since this requires actively seeking patient consent, educating patients about the use of AI in their care, and preserving their faith in the medical system [132,133].

Multimodal AI has great potential to improve healthcare outcomes; nevertheless, many obstacles must be overcome before these technologies can be fully utilized in clinical settings. Cooperation between researchers, clinicians, legislators, and industry stakeholders is crucial to integrating multimodal AI in clinical practice. Addressing these limitations involves ongoing research and collaboration between researchers, engineers, clinicians, and healthcare professionals to develop robust frameworks of multimodal AI in cancer care.

## 5. Future Directions

The future development of multimodal AI can pave the way for improvements in personalized medicine, general oncology, and the healthcare system.

### 5.1. Cancer Patient Digital Twin

The high-dimensional properties of omics data and AI can be used to create more complex systems that can replicate patient responses in real time [5,134]. The virtual replica of a patient’s cancer, called the cancer patient digital twin (CPDT), combines extensive data such as genetic information, molecular traits, spatial heterogeneity, and dynamic behavior across time. The CPDT dynamically changes as the patient’s tumor advances, reflecting the patient’s response to tumor progression and therapy [135]. The critical components of the CPDT rely on the available longitudinal data, which fuel the virtual twin to mimic trends and patterns over an extended period, enabling more accurate patient monitoring and informed decision making. This holistic approach not only supports clinicians in adjusting personalized treatment plans but also supports patients by providing them with comprehensive insights into their disease trajectory. Currently, several pilots have been launched to guide treatment in several fatal cancers such as pancreatic, melanoma, lung, and breast cancers [136].

### 5.2. Virtual Clinical Trial

While clinical trials are indispensable in diagnostic, prognostic, and therapeutic validations, randomized clinical trials are often costly, time consuming, and limited by the number of participants [137,138,139]. These constraints pose clear challenges to evaluate novel treatment and therapies in a timely manner, therefore delaying their availability to patients in need. By leveraging ML algorithms, multimodal AI can compensate for the limited participant enrollment through the generation of digitizing cohorts based on multiple sources. For example, retrospective studies from small cohorts can serve as a foundation for training and validating ML models to create a large virtual cohort [139]. The digital clinical trial not only improves biomedical phenotyping, especially for underrepresented populations, but also provides the opportunities to modify trials in real time for better detection, decreasing risks, and management of adverse events.

### 5.3. Healthcare System

Multimodal AI data integration can greatly improve the health system. In healthcare, staff shortages are a significant challenge, leading to increased workloads and potential burnout for existing staff [140]. By creating a standardized workflow combining important patient data into a cohesive system, this integration can automate repetitive and time-consuming tasks such as data entry, appointment scheduling, and patient triage. For example, automation can help in (i) reducing manual errors, (ii) ensuring that critical information is readily accessible, and (iii) enhancing routine tasks. As a result, healthcare professionals will be able to manage their time between tedious tasks and patient care [141].

For patients under continuous evaluation, AI systems can efficiently manage the collection of clinical data from various sources, such as hospital equipment and in-home health monitors, to identify trends and abnormalities. During consultations, these systems can provide healthcare providers with comprehensive insights into a patient’s condition, including potential diagnoses and treatment options. In this context, AI-based monitoring can be seen as a virtual assistant in early detection and treatment, allowing proactive intervention in the treatment plan to address any associated side effects. This proactive approach can lead to better patient outcomes and more efficient use of healthcare resources [5].

## 6. Conclusions

In conclusion, multimodal AI represents a significant advancement in precision medicine, through the combination of diverse modalities, to create personalized treatments and enhance patient care. Based on the examples provided in this review, multimodal AI holds the potential to foster a comprehensive understanding of cancers and their complex ecosystem. The integration of diverse biomedical data could mitigate uncertainties and optimize diagnosis, prognosis, and treatment protocol adjustments. With ongoing advancements in AI and big data analytics, integrating multimodal AI into oncology practice is increasingly viable, promising substantial advancements in managing and treating challenges in the case of B-NHLs. Moreover, these technological advances can revolutionize healthcare by improving patient outcomes and decision making, not only in lymphoma but across complex and incurable cancers.
